# Durability of Nano-Reinforced Recycled Aggregate Concrete under Load and Chloride Ingress

**DOI:** 10.3390/ma15186194

**Published:** 2022-09-06

**Authors:** Yongdong Yan, Youdong Si, Yulong Zheng, Xin Wang

**Affiliations:** Faculty of Civil Engineering and Mechanics, Jiangsu University, Zhenjiang 212013, China

**Keywords:** recycled aggregate concrete, chloride ion, bending load, steel corrosion, concrete durability

## Abstract

The improved performance of recycled aggregate has an important impact on its use in engineering. In this study, to improve the weak surface properties, recycled aggregates were treated by nano-silica slurry and applied to concrete beam specimens. Under the action of cracks caused by continuous load and drying-wetting cycles with chloride ingress, the effects of different recycled aggregate additions, nano-silica contents and crack widths on the self-healing performance of cracks and the resistance to chloride ingress of the recycled concrete beams were investigated. It was found that the self-healing rate of cracks increased first and then decreased with increased nano-silica content, reaching a maximum when the content reached 0.4%. Greater amounts of additive in the recycled aggregate increased the concentration of free chloride ions in cracks. However, this concentration was found to be weakened in nano-reinforced aggregate. From a comprehensive perspective, the relative chloride ion concentration can be effectively reduced by controlling the crack width to be smaller than 0.12 mm and using improved recycled aggregates treated with 0.2% nano-silica material. This study provides a reference for the application of recycled aggregate concrete under severe environmental and load conditions.

## 1. Introduction

The large amount of construction waste generated by the demolition of buildings exerts great pressure on the environment [1,2]. At present, construction waste is mainly landfilled or piled up for treatment [3], which is harmful to the surrounding ecological environment. In order to solve this problem, the concept of recycled aggregate concrete (RAC) using construction waste came into being [4,5].

However, there are still some problems in the practical application of RAC. Peng et al. [6] found that in RAC beams, the diffusion rate of chloride ions was accelerated with a higher replacement rate of recycled aggregate, increasing the risk of reinforcement corrosion and reducing the bearing capacity of concrete beams. This is mainly because the recycled aggregate has a complex interface transition zone [7,8], and initial cracks may appear in the crushing process, causing the physical and mechanical properties of recycled aggregate concrete to decline to a certain extent. In addition, for concrete structures including RAC that serve in chloride-rich environments such as coastal areas, the concrete surface is prone to cracking, which provides a further path for the diffusion of harmful ions (such as SO_4_^2−^ and Mg^2+^, mainly Cl^−^), especially in alternating wet and dry areas like splash and tidal zones. Thus, the diffusion of harmful ions will be accelerated, which induces reinforcement corrosion. In severe cases, the protective layer might be peeled off, further deteriorating the durability of concrete structures.

In this regard, research on improving the performance of RAC, especially in terms of its durability, have been carried out. Researchers have studied the effects of adding steel fiber and fly ash [9], applying treatment combining spraying calcium rich wastewater and flow carbonization [10], fly ash sulfate activation [11], the addition of micro carbon fiber (CF) [12] and combining mechanical treatment and silica fume [13]. It has been proven that the mechanical properties and partial durability of RAC can be improved by using nano-strengthened slurry treatment. At a microscopic level, Li et al. [14] found that the nano-silica treatment of recycled aggregate can improve the micro hardness of the old and new mortar near the interface. Additionally, Xiao et al. [15] proposed a dynamic stress–strain constitutive model of the modified RAC. It was found that recycled aggregate treated with nano-silica can achieve relatively better performance compared to the aforementioned methods [16], making it a potential method to promote the utilization of recycled aggregates.

At present, scholars are mostly studying the basic material performance of recycled aggregates containing nano-silica slurry [17,18]. However, in many cases, concrete components need to work under the coupling effect of continuous load-induced cracking and erosive environments, which may influence effects of nano-silica. Therefore, in order to promote the application of RAC, the performance of RAC structural components made from recycled aggregate and treated with nano-silica slurry under external load and environmental effects needs to be further studied.

This study focuses on the effect of nano-silica treatment on the performance of beam specimens made of treated recycled aggregate under the coupling action of load and chloride ingress. The distribution of chloride ions at or near the cracks induced by external load, as well as the corrosion behavior of steel bars, are discussed in this paper, considering the influence of the content of nano-silica, the replacement rate of recycled aggregates, and the width of the cracks. On this basis, the apparent chloride ion diffusion coefficients at or near the cracks were analyzed by regression analysis. This study provides a reference for durability analyses and life predictions, and seeks to promote the broader application of RAC under severe conditions.

## 2. Materials and Methods

### 2.1. Materials

**Figure 1:**
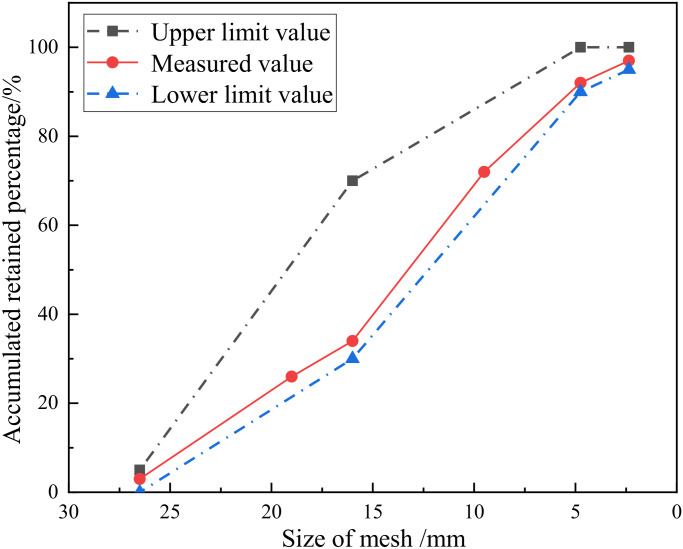
Sieving curve of recycled aggregate.

Ordinary Portland cement (P·O 42.5) produced by Jiangsu Helin was adopted; its properties met the relevant standards [19], as shown in Table 1. The applied natural coarse aggregate comprised 5~25 mm continuous graded crushed stone. The recycled aggregates came from a demolished building at Jiangsu University, which had been used for 20 years. After being selected, crushed and screened, the recycled aggregates were mixed and then tested according to the specifications regarding “Recycled coarse aggregate for concrete” (GB/T 25177-2010) [20]. The sieving curve of recycled aggregate is shown in Figure 1, and the specific properties are shown in Table 2. The main properties of the applied nano-silica are given in Table 3. The fine aggregate was river sand, with a fineness modulus of 2.7. Tap water was used to improve the workability of the RAC. The mix proportion and compressive strength of the recycled aggregate concrete is shown in Table 4. In the test, three specimens measuring 150 mm × 150 mm × 150 mm were prepared for each group according to the “Standard for test methods of concrete physical and mechanical properties” (GB/T 50081-2019) [21].

### 2.2. Preparation of Nano Reinforced Recycled Aggregate

**Figure 2:**
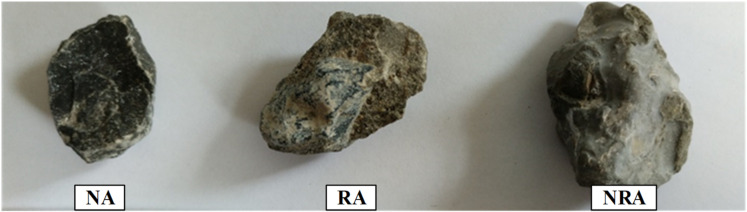
Surface morphologies of natural aggregate (NA), recycled aggregate (RA) and nano recycled aggregate (NRA).

Considering that nano-silica is poorly soluble in water, and clusters of insoluble substances formed, a 230 T ultrasonic disperser was used. After that, cement was added to the solution at a ratio of 0.5 by fully mixing; in this way, the nano reinforced slurry was made. For each treatment, 5 kg of recycled aggregates was put into 10 L of nano reinforced slurry and stirred for 3 min. These mixtures were then left to stand for 30 min before being taken out and dried in air for 24 h. Images of the aggregates before and after strengthening are shown in Figure 2.

### 2.3. Self-Anchored Crack

**Figure 3:**
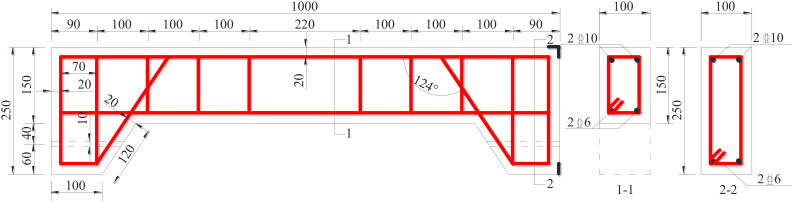
Schematic diagram of test beam member (unit: mm).

A total of eight RAC beams were manufactured, with dimensions of 100 mm × 150 mm × 1000 mm. As described in Table 4 and Table 5, in these specimens, the following parameters were controlled: the replacement rate of recycled aggregate, the nano-silica content and the maximum crack width (detailed later). These variables were expected to influence the chloride ingress effect in the RAC component. HRB400 steel bars with diameter of 10 mm were adopted as longitudinal bars; the thickness of the protective layer was 20 mm. HPB300 steel bars with diameter of 6 mm were utilized for the stirrups. The performance of the steel reinforcements complied with the national standards [22,23]. The spacing between the stirrups at both ends was 50 mm, and at the middle of the span was 220 mm. Holes 10 mm in diameter were made at both ends of the specimen for loading with a threaded rod (detailed later). Details regarding the specimens and reinforcements are provided in Figure 3.

**Figure 4:**
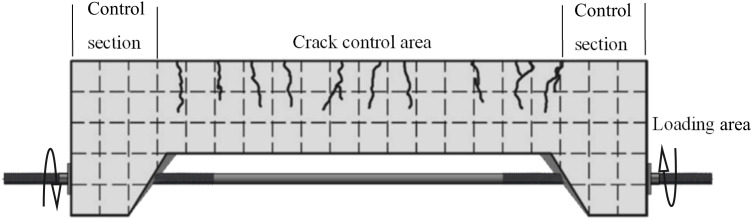
Schematic diagram of test beam loading.

The test beam was loaded onto a threaded rod with a diameter of 10 mm, which was inserted into the reserved hole, and a gasket was placed at each end of the nut to prevent local damage due to stress concentration. When loading, the threaded rod was symmetrically tensioned by a torque wrench in stages. Cracks of different widths were then generated in the control area of the test beam. In line with “Code for design of concrete structures”(GB50010-2010) [24], the maximum crack width of reinforced concrete structures under various environments was limited to 0.1~0.4 mm. For stricter conditions, the maximum control crack width was limited to 0.1~0.3 mm. In this experiment, the crack width was controlled using a crack width meter with an accuracy of 0.01 mm. To facilitate measurement, lime was smeared on the side of the beam before loading, and 50 mm × 50 mm grids were drawn. A schematic diagram of the specimen is shown in Figure 4.

The statistical results of the self-anchored cracks of the specimen are shown in Table 5, from which it can be determined that:

- The maximum crack width can be effectively controlled to reach the intended value through the stepped loading method using a stress wrench.
- With an increase in the maximum crack width, although the total number of cracks increased, the average spacing value showed a downward trend.
- The average spacing of cracks was approximately proportional to the replacement ratio of recycled aggregate.

### 2.4. Chloride Drying-Wetting Cycle

**Figure 5:**
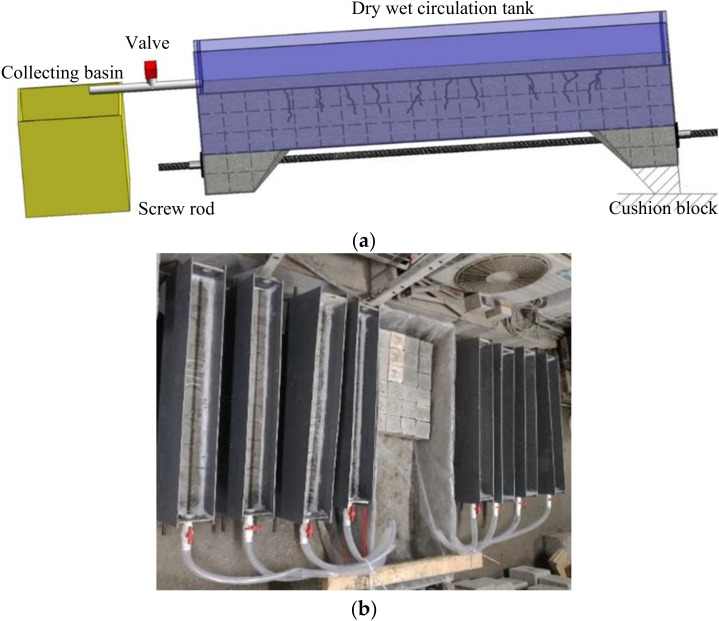
Design (**a**) and photo (**b**) of the setup.

After the test beam was loaded, the drying-wetting cycle experiment was carried out. The design and physical setup of the system, comprising the test beam to be corroded, the corrosion tank and a collecting basin, are shown in Figure 5a,b respectively. The following factors were considered:

- The corrosion tank, independently installed on the beam, was arranged along the general length of the member and used a PVC plate with high corrosion resistance as raw material. An epoxy resin sealant was coated on each of the lateral sides of the beams.
- In order to facilitate the drying-wetting cycles, a valve was installed at the bottom of one side of the corrosion tank, and a cushion block was placed on the other side of the beam. In this way, the collecting basin could be filled with chloride solution when the valve was opened.

A NaCl solution of 5% mass concentration was adopted as the corrosion solution. The time ratio of the drying-wetting cycle was 1:1, with each cycle period being 6 days for a total of 50 cycles. In order to control the mass concentration, a densitometer was installed in the container. The NaCl solution was replaced when the mass concentration changed.

### 2.5. Chloride Ion Content Detection

**Figure 6:**
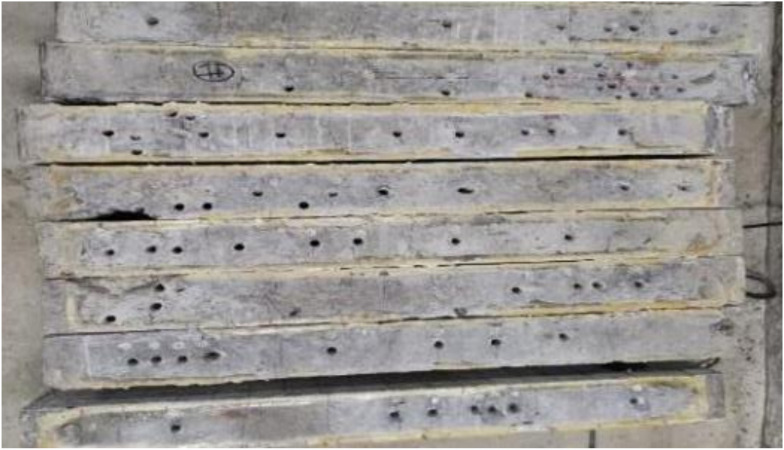
Distribution of detection points.

After the drying-wetting test, the water tank and the loading device were removed. At this point, the specimens were placed outdoors. When the test beam was completely air-dried, an impact drill with a diameter of 14 mm was used to extract samples from a crack and from other positions (i.e., 30, 60 and 90 mm from the crack). Samples were taken at intervals of 5 mm in the depth direction in order to obtain concrete powder from different regions. The distribution of detection points for powder collection is shown in Figure 6. Each powder sample was then put into an aluminum box and dried in an oven (100 ± 5 °C), Next, 1.5 g of each powder sample was added to 10 mL of distilled water and placed in an ultrasonic bath for 5 min. Subsequently, bottles were opened and left to stand for 24 h to obtain the solutions for testing. Finally, the Rapid Chloride Test (RCT) [25] electrode, wetted one day in advance, was used to determine the free chloride ion contents in the solutions.

## 3. Results

To evaluate the performance of reinforced RAC under continuous load and erosive environments, the influences of crack width, nano-silica content and replacement ratio of recycled aggregate on self-healing and anti-chloride ingress capacity were investigated.

### 3.1. Self-Healing of Transverse Cracks

#### 3.1.1. Morphology of Transverse Cracks

**Figure 7:**
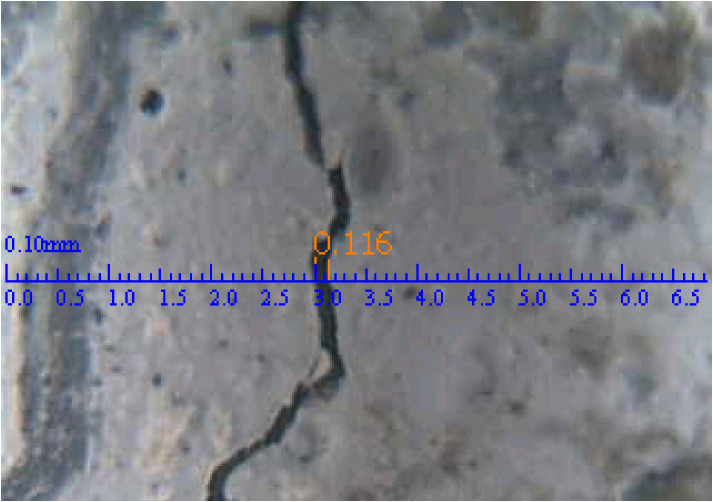
Typical crack width measurement result (unit: mm).

The transmission of chloride ions is affected by the width and development of cracks in the process of self-anchoring. Therefore, the crack widths of each component at different times after loading were detected using a crack width meter, with one typical detection result shown in Figure 7.

**Figure 8:**
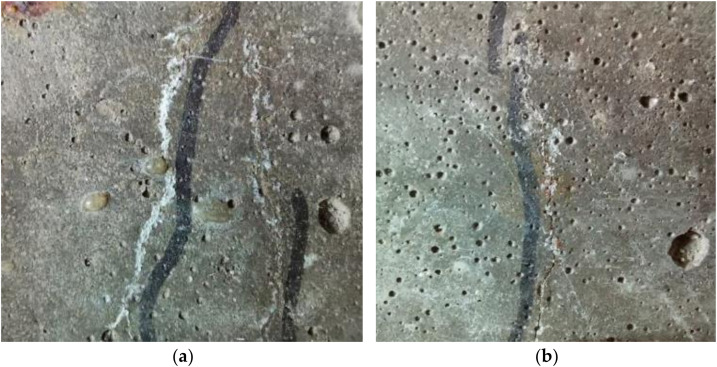
Completely self-healed crack (**a**) and partially self-healed crack (**b**).

From a statistical analysis of the measured crack width results from different periods, it was found that:

- After 300 days of drying-wetting cycles, although the crack width slightly decreased, the loaded test beam maintained a cracking state. After unloading, the width of the crack decreased sharply, indicating that the self-anchoring method of the specimen had provided an effective continuous load to maintain the crack width.
- When *w* (crack width) ≤ 0.075 mm, 7.6% of the cracks were completely healed; this was probably because the self-healing substances, such as the CaCO_3_ produced by the hydration of unreacted cement particles, were filling in and healing the cracks. In addition, nano-silica particles could fall off the aggregate surface, filling the internal pores of the concrete and generating a C-S-H gel due to a pozzolanic reaction, which further filled in the cracks and promoted the self-healing effect [26,27]. An example of the crack healing is shown in Figure 8a.
- When 0.075 mm < *w* < 0.158 mm, the width of the crack was reduced to varying degrees after the drying-wetting cycles. On the one hand, this may have been due to the self-repairing effect of the concrete material itself, which reduced the width, as shown in Figure 8b. On the other hand, it may have been caused by the decrease of stress in the threaded rod over time, i.e., the creep effect which further weakened the anchoring load.

#### 3.1.2. Self-Healing Rate of Transverse Cracks

Through a comparative analysis before and after the drying-wetting cycles, it was found that the change of crack width in the test beam was affected by the maximum control width of the crack, the aggregate substitution rate and the nano-silica content. Therefore, the self-healing rate could be calculated using the following formula:

(1) $$\omega =\frac{w_{1}-w_{2}}{w_{1}}\times 100\%$$

where *ω* is the self-healing rate of cracks, *w*_1_ is the crack width (mm) before the drying-wetting cycles and *w*_2_ is the crack width (mm) at the end of drying-wetting cycles. The sample standard deviation of the crack self-healing rate, represented by *σ*, was computed as follows:

(2) $$\sigma =\sqrt{\frac{1}{N-1}\sum_{i=1}^{N}{\left(\omega_{i}-\mu \right)}^{2}}$$

where *N* is the number of cracks, *ω_i_* is self-healing rate corresponding to different crack widths and *μ* is the arithmetic average value of the self-healing rate for different crack widths.

**Figure 9:**
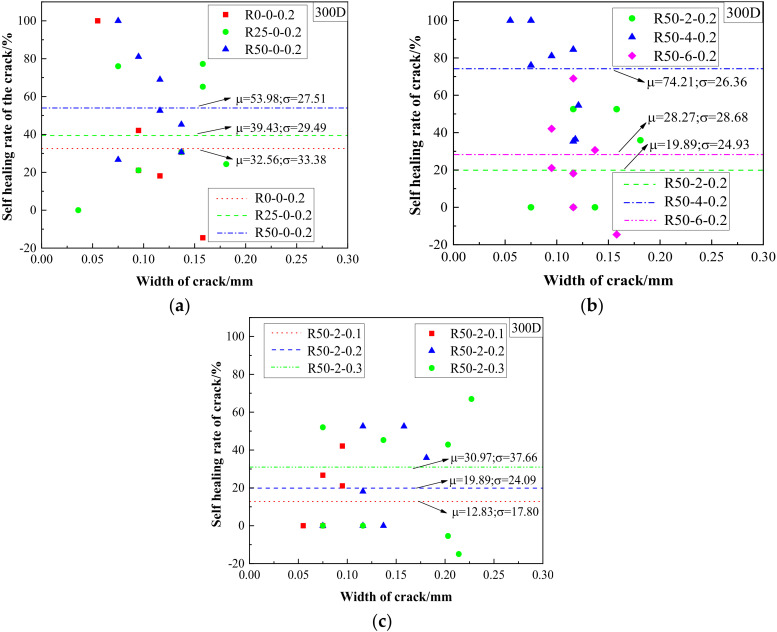
Self-healing rate of cracks in R0-0-0.2, R25-0-0.2 and R50-0-0.2 (**a**); R50-2-0.2, R50-4-0.2 and R50-6-0.2 (**b**); R50-2-0.1, R50-2-0.2 and R50-2-0.3 (**c**).

The self-healing rate is shown in Figure 9, from which, the following conclusions can be drawn:

- As shown in Figure 9a, with an increase in the replacement rate of recycled aggregate, the self-healing rate of cracks gradually improved, and the standard deviation was reduced, indicating that the addition of recycled aggregate is beneficial to the self-healing ability of concrete.
- As shown in Figure 9b, with an increase in the nano-silica content, the self-healing of cracks first increased and then decreased, reaching a maximum when the content of nano-silica was 0.4% [26,27,28]. In addition, the nano-silica soaking method was proven to effectively heal defects in the recycled aggregate and to compensate for the strength loss of the aggregate, mainly because the nano-silica particles react with the calcium hydroxide hydration product to form C-S-H gels on the aggregate surface [15,29]. The formation of C-S-H gel can be effectively promoted by using the proper amount of nano-silica to fill the pores of concrete [30].
- As shown in Figure 9c, as the controlled crack width increased, the self-healing ability improved gradually. This may have been due to the fact that sufficient water was provided to the interior of the concrete through larger cracks, which promoted later hydration reactions in the cement, thereby helping to promote the generation of hydration products and filling the cracks to improve the self-healing rate.

In summary, the nano-silica slurry treated method improves the self-healing capacity of cracks. With the increase of nano-silica content, the self-healing rate of the cracks increases first and then decreases. When the nano-silica content is 0.4%, the self-healing rate is optimal.

### 3.2. Distribution of Chloride Ions

#### 3.2.1. Distribution of Chloride Ions in Cracks

Regarding the beam specimens shown in Table 5, about eight cracks with different widths were formed on each specimen surface after loading. In order to explore the effect of the actual crack widths on chloride ion concentrations, the crack width values shown in the following content refer to the actual width of the cracks selected in each beam.

**Figure 10:**
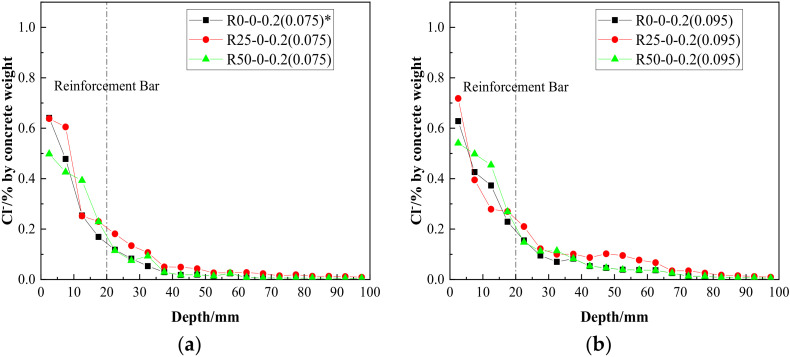

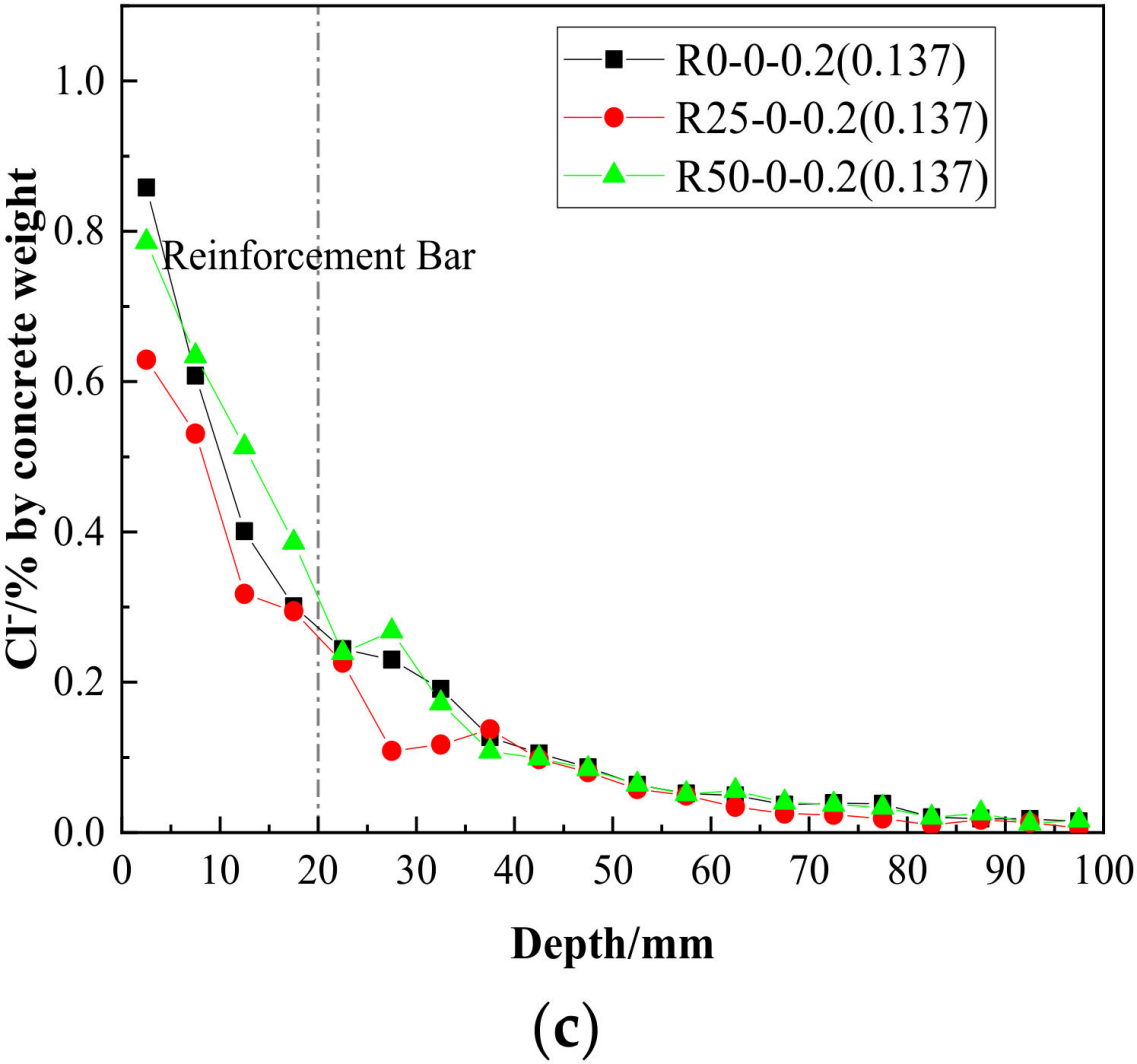
Distribution of chloride ion contents at different depths as a function of aggregate substitution when *w* = 0.075 mm (**a**), *w* = 0.095 mm (**b**) and *w* = 0.137 mm (**c**). Note (*): the values in brackets refer to the actual widths of the cracks selected in the specified beam.

Assuming a constant crack width, the chloride ion concentration results at different depths from the surface of the RAC beams, along with the replacement rates of the recycled aggregate, are illustrated in Figure 10. It can be seen that:

- The chloride ion content showed a wave-decreasing trend according to depth [31], which may have been due to the fact that larger aggregate particles are encountered at certain depths; the concentrations of chloride ions in coarse aggregate were far lower than in the cement mortar.
- As shown in Figure 10a, at the position of longitudinal reinforcement, the chloride ion concentration of the natural aggregate concrete in a crack of 0.075 mm was 0.14%. A lot of research [32,33] has shown that the critical concentration for reinforcement depassivation is 0.1–0.6% in cementitious materials. Since cementitious material made up 15.07% of the concrete mass, the critical concentration was about 0.015–0.09%, which indicated that the reinforcement had been activated.
- For beams with recycled aggregate replacement ratios of 25% and 50%, the concentrations of free chloride ions at the longitudinal reinforcements were 1.5 and 1.2 times those in beams with natural aggregates (crack width: 0.075 mm; see Figure 10a), which infers that the existence of recycled aggregate increases the concentration of free chloride ions at cracks. However, as shown in Figure 10c, with the increase of crack width, the chloride ion concentration in the concrete with a 25% replacement ratio was gradually exceeded by that of concrete with a 50% ratio; this was probably because that the crack had a greater impact on the chloride ion corrosion resistance in the RAC with a higher replacement rate.
- From a comparison of Figure 10a–c, it can be seen that the distribution trend of chloride ions at the three groups with different crack widths was almost the same. However, with an increase in crack widths, the total free chloride ion concentrations increased.

**Figure 11:**
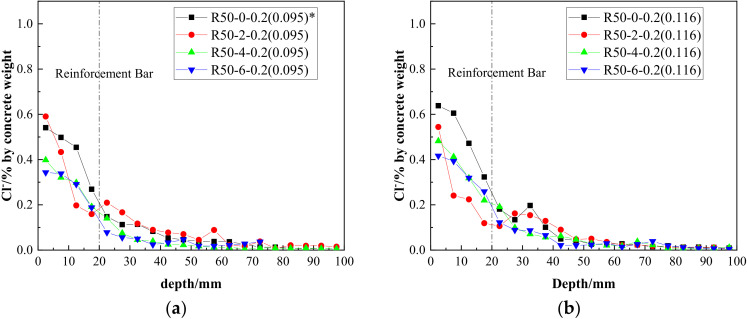

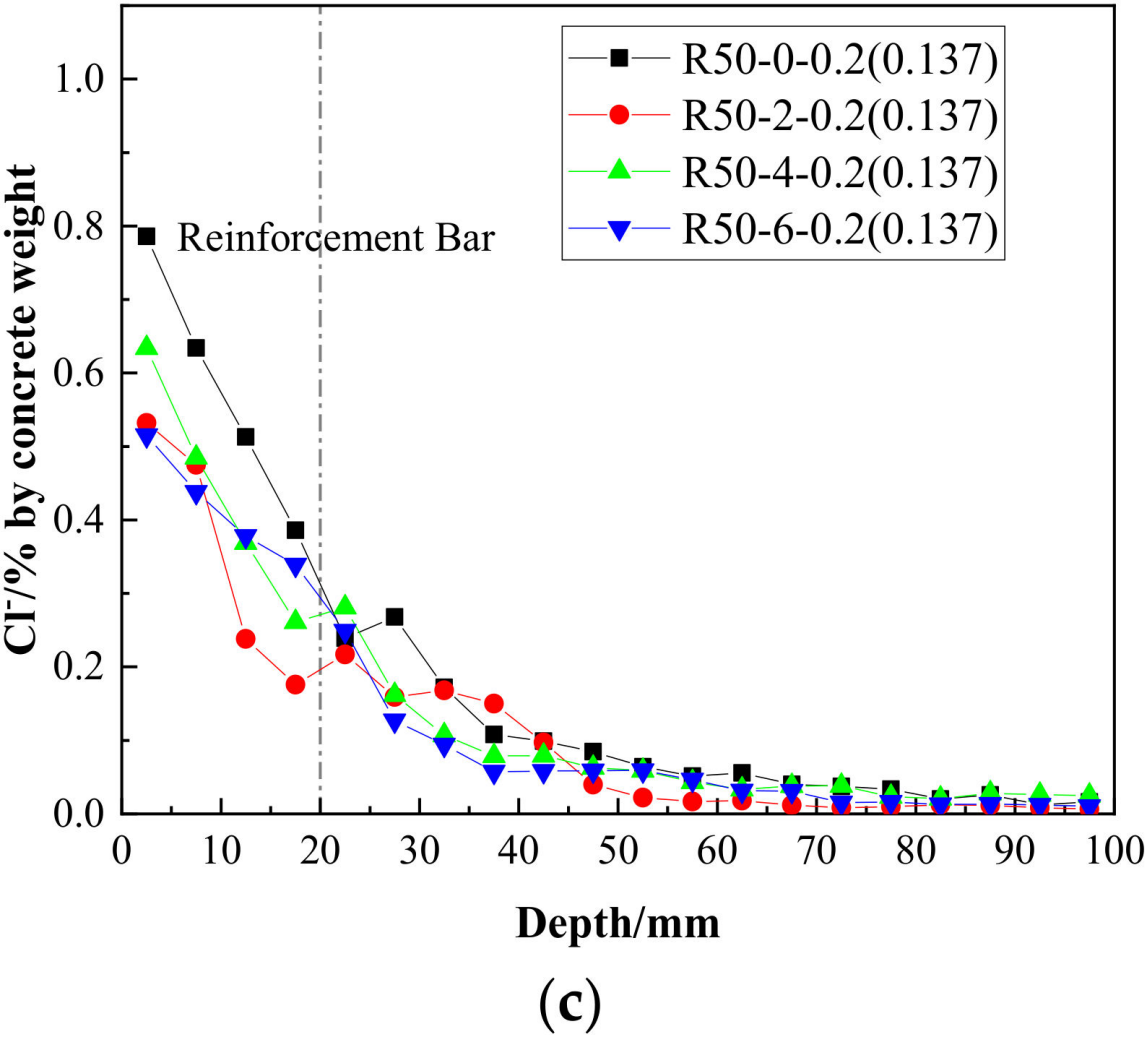
Distribution of chloride ion content at different depths as a function of nano-silica content when *w* = 0.095 mm (**a**), *w* = 0.116 mm (**b**) and *w* = 0.137 mm (**c**). Note (*): the values in brackets refer to the actual widths of the cracks selected in the specified beams.

Assuming a constant replacement rate of recycled aggregate, the concentration of chloride ions at different crack depths, along with the variation of the nano-silica content, are shown in Figure 11. It can be concluded that:

- Notwithstanding a small amount of fluctuating data, by adding nano-silica, the chloride ion concentrations at the reinforcement bar position decreased at different crack widths, confirming the effectiveness of nano-silica at resisting chloride diffusion.
- The trend of wave-decreasing along the depth was not changed by the addition of nano-silica at the crack of the RAC beams.
- From a comprehensive comparison of Figure 11a–c, it may be seen that the concentration of free chloride ions at cracks could be effectively reduced by nano-silica modification. When the crack width was 0.095 mm and the concentration of nano-silica reached 0.6%, the reduction effect was the most obvious.
- Comparing Figure 11a–c, it can be seen that the optimal content of nano-silica changed with different crack widths; however, this needs to be further studied considering the width, type and curvature of the cracks.

**Figure 12:**
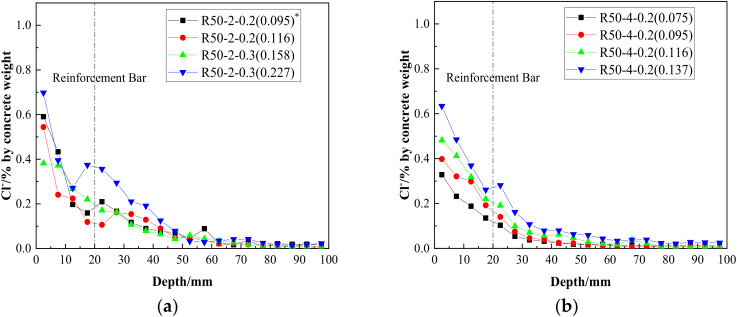

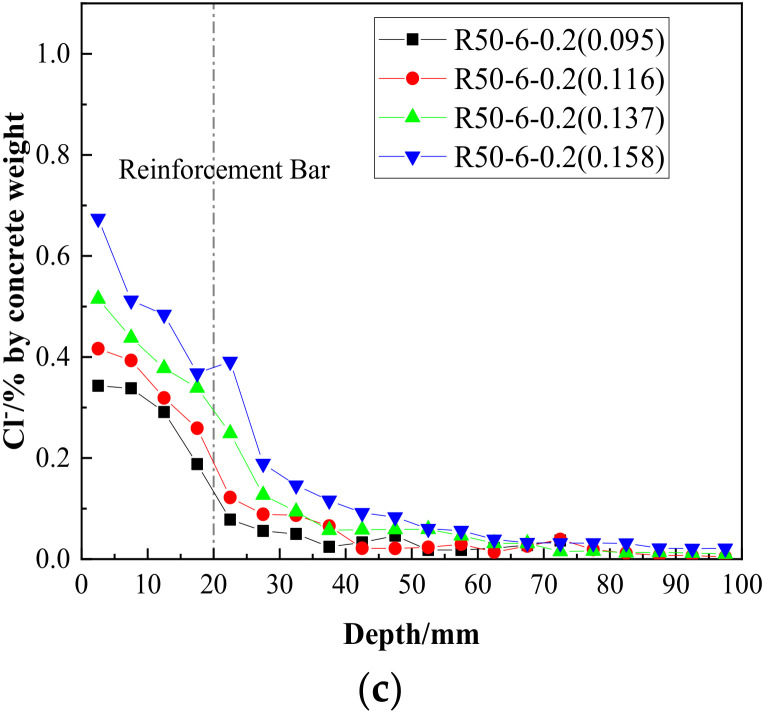
Distribution of chloride ion contents at different depths as a function of crack width when f*^2^ = 0.2% (**a**), *f* = 0.4% (**b**) and *f* = 0.6% (**c**) (the mass percentage of nano-silica in the cementitious material). Note (*): the values in brackets refer to the actual width of the cracks selected in the specified beams.

Assuming a constant replacement rate of recycled aggregate, the concentrations of chloride ions at different crack depths and the variation of crack widths are presented in Figure 12, from which, it can be determined that:

- With an increase of crack width, the concentrations of free chloride ions in the nano-reinforced RAC beam increased gradually; this phenomenon was more clearly observed with increased content of nano-silica.
- There was no obvious regularity regarding the concentration of chloride ions in the range of 5 mm with different cracks as the transportation of chloride ions on the concrete surface was affected by convection, diffusion, surface adsorption and capillarity. Since these mechanisms are relatively complex, the equivalent chloride diffusion coefficients within 5 mm were not considered in this study.
- By comparing Figure 12a–c, it can be found that the concentrations of free chloride ions at the reinforcement position increased sharply with an increase of crack width. Among them, in the RC beam with a nano-silica content of 0.2%, when the crack width was 0.227 mm, the concentration of free chloride ions was about double that of a crack width of 0.095 mm.

#### 3.2.2. Distribution of Chloride Ion Concentration near Cracks

**Figure 13:**
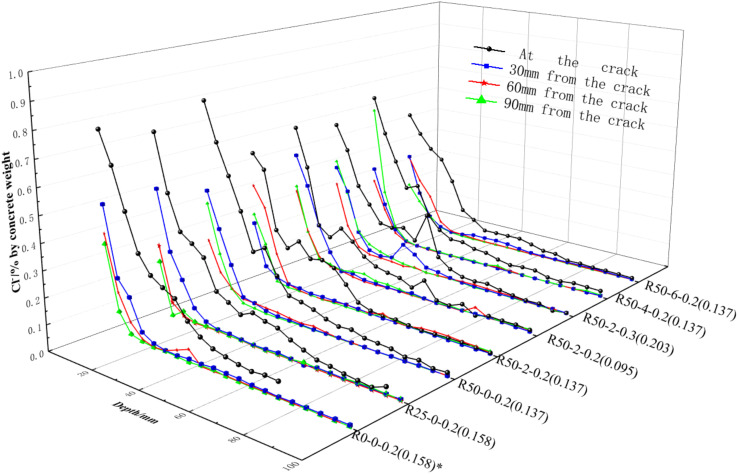
Distribution of chloride ion contents at different depths as a function of distance. Note (*): the values in brackets refer to the actual widths of the cracks selected in the specified beams.

The concentrations of chloride ions within a certain range near cracks are shown in Figure 13. As shown in the figure:

- The concentrations of chloride ions at cracks were significantly higher than those near the cracks. This was mainly because the permeability of the concrete had been improved due to the presence of cracks. In addition, near larger cracks (i.e., width more than 0.1 mm), chloride ions presented a trend of two-dimensional diffusion [34] i.e., diffusion along the depth of concrete and lateral diffusion near the crack.
- The concentrations of chloride ions showed a wavy declining trend along the depth direction. With an increase of crack width, the fluctuation of chloride ion concentrations also increased, which was similar to the micro simulated results [35] of chloride ion diffusion in RAC based on a random distribution of aggregate.

#### 3.2.3. Distribution of Relative Chloride Ion Concentrations on Longitudinal Reinforcements

**Figure 14:**
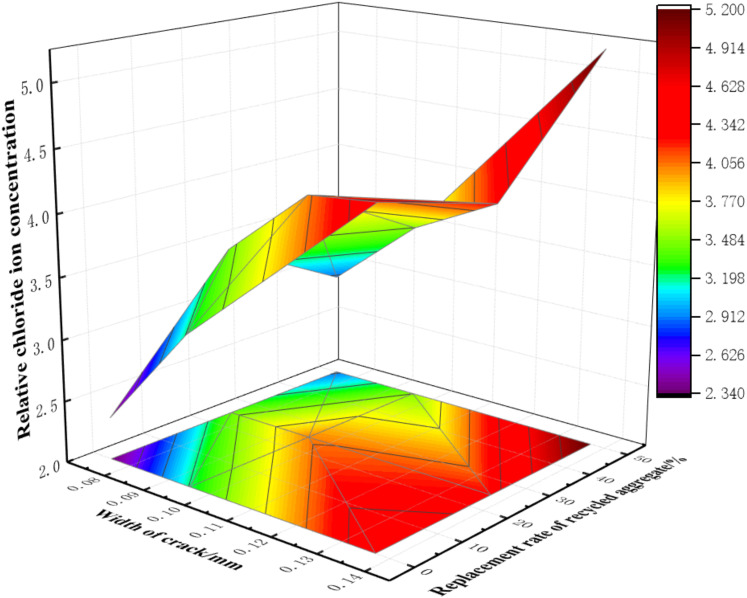
Distribution of relative chloride ion concentrations along with variations of crack width and replacement rate of recycled aggregate.

In order to further analyze the distribution of chloride ion concentrations on the longitudinal bars of nano-reinforced RAC beams, the concentrations at various depths are shown in Figure 11, Figure 12 and Figure 13. Additionally, the distribution of relative chloride ion concentrations, along with the change of crack width and the replacement rate of recycled aggregate, was studied; the analysis results are shown in Figure 14. As can be seen:

- The relative concentrations of chloride ions at cracks were greater than 1.0 under the action of drying-wetting cycle, which showed that the diffusion rate of chloride ions could be effectively accelerated by the test method described in this paper. Additionally, the relative concentrations of chloride ions increased steadily with the increase of crack width.
- The relationship between the concentration of relative chloride ions and the replacement rate of recycled aggregate was limited by the crack width (*w*): when *w* ≤ 0.1 mm, it increased first and then decreased with an increase in the replacement rate of recycled aggregate; the maximum value was reached when the replacement rate reached 25%. When 0.1 mm < *w* ≤ 0.135 mm, with the increase of replacement rate, it first increased, then decreased, and finally, increased again, with the optimal replacement rate being about 35%. When *w* > 0.135 mm, the effect of aggregate replacement rate was almost negligible.

**Figure 15:**
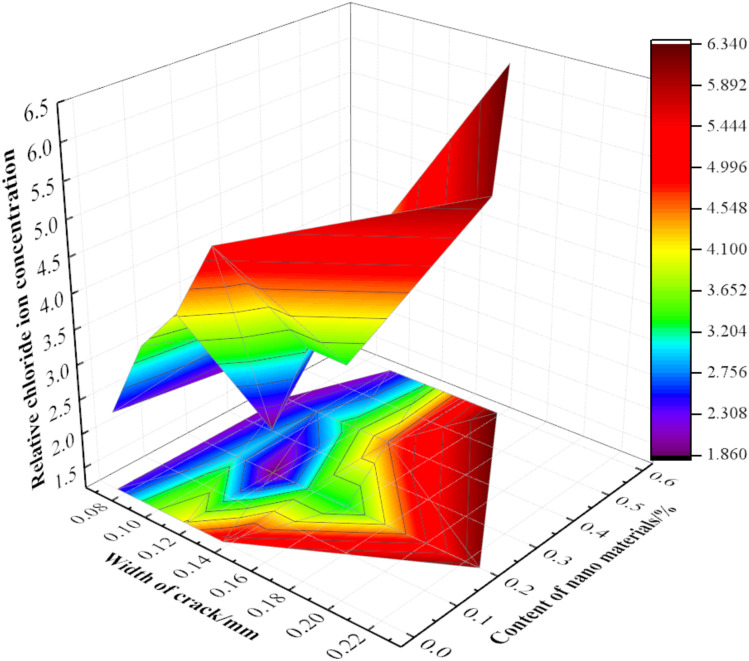
Distribution of relative chloride ion concentrations with crack width and nano-material content.

With a replacement rate of recycled aggregate of 50% at the position of the longitudinal bars, the analysis results of changes in the relative chloride ion concentrations with varying crack widths and contents of nano-silica are shown in Figure 15. It can be seen that:

- The width (*w*) of the cracks was a key factor affecting the concentrations of chloride ions.
- To a certain extent, the concentration of relative chloride ions could be reduced by nano modification technology; this may be because the binding capacity of recycled concrete to chloride ions is improved by the modified recycled aggregate to some degree [11,36,37].
- Although the concentration of relative chloride ions could be reduced by nano modifications, it is still restricted by crack width: when *w* ≥ 0.185 mm, the concentration of relative chloride ions was almost unaffected by the nano-silica content. When 0.14 mm ≤ *w* < 0.185 mm, the concentration of relative chloride ions first decreased and then increased with an increase of nano-silica content. When the content of nano-silica was 0.2%, the concentration of chloride ions at cracks in the modified recycled concrete reached the lowest value. When 0.1 mm ≤ *w* < 0.14 mm, the relative chloride concentrations first decreased, then increased, and finally, decreased again according to the nano-silica content. The optimal content of nano-silica was found to be in the range of 0.15–0.3%, although the higher contents need further experimental research. When 0 ≤ *w* < 0.1 mm, the concentration of relative chloride ions increased with an increase of nano-silica content.
- When cracks appeared in the RAC, the relative chloride concentrations could be effectively reduced by controlling the crack width *w* ≤ 0.12 mm and adding about 0.2% nano-silica modified recycled aggregate. As such, further the economic benefits could be obtained.

Therefore, the nano-silica treatment method can improve the resistance of cracked RAC to chloride ingress to a certain extent. The relative chloride ion concentration can be effectively reduced when the crack width is smaller than 0.12 mm and the nano-silica content is 0.2%.

### 3.3. Calculation and Analysis of Relative Apparent Chloride Diffusion Coefficient

Once the equivalent diffusion coefficient of chloride ions at cracks in the nano-reinforced RAC beams had been calculated, the nonlinear curve fitting toolbox in the Origin software was used to carry out a regression analysis on the data in Figure 12 and Figure 13, and the Levenberg-Marquardt algorithm was used to carry out optimization calculations. The most noteworthy observations were as follows:

- The value of the sudden change points in chloride ion concentrations had to be discarded in the regression analysis.
- Because the extracting depth of the powder was large and the data were sufficient, the missing data could simply be ignored.
- As the replacement rate of recycled aggregates and the amount of nano-silica changed, the fitting parameters needed to be adjusted appropriately.

**Figure 16:**
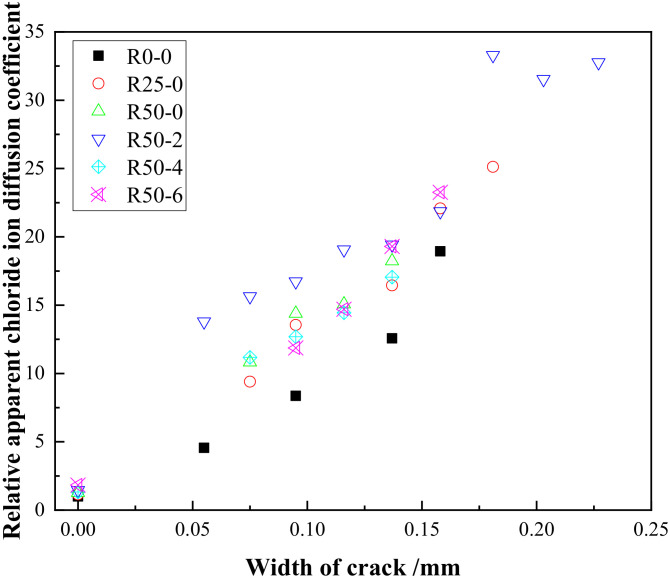
Relative apparent chloride diffusion coefficient at each crack after 50 wetting-drying cycles.

After 50 drying-wetting cycles, the equivalent chloride ion diffusion coefficients at the crack were calculated and fitted. The chloride ion diffusion coefficient at the uncracked part of natural aggregate concrete was taken as the benchmark (1.09 × 10^−12^ m^2^/s) to calculate the relative coefficients under different working conditions. The results are shown in Figure 16. As can be seen:

- The relative apparent chloride diffusion coefficients of natural aggregate concrete showed an upward trend with the increase in crack width [38].
- When the modified recycled aggregate was not considered, the chloride diffusion coefficients of ordinary RC beams increased with an increase in the replacement rate of recycled aggregate, and the chloride corrosion resistance of RAC was reduced due to the presence of recycled aggregate [39,40].
- When the crack width was greater than 0.15 mm, there was a sudden change in the relative apparent chloride diffusion coefficient. The reason for this phenomenon needs to be further analyzed, in combination with the chloride ion diffusion mechanism at the crack.

**Figure 17:**
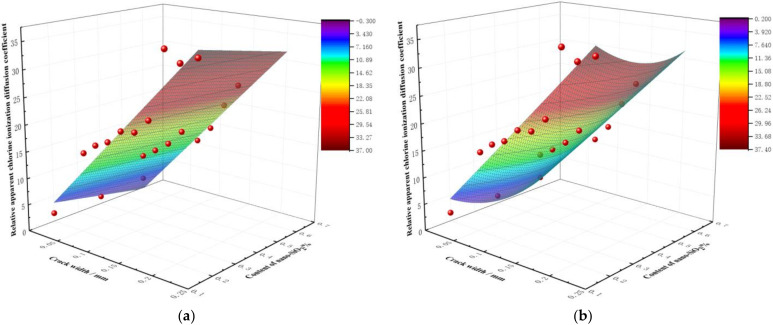

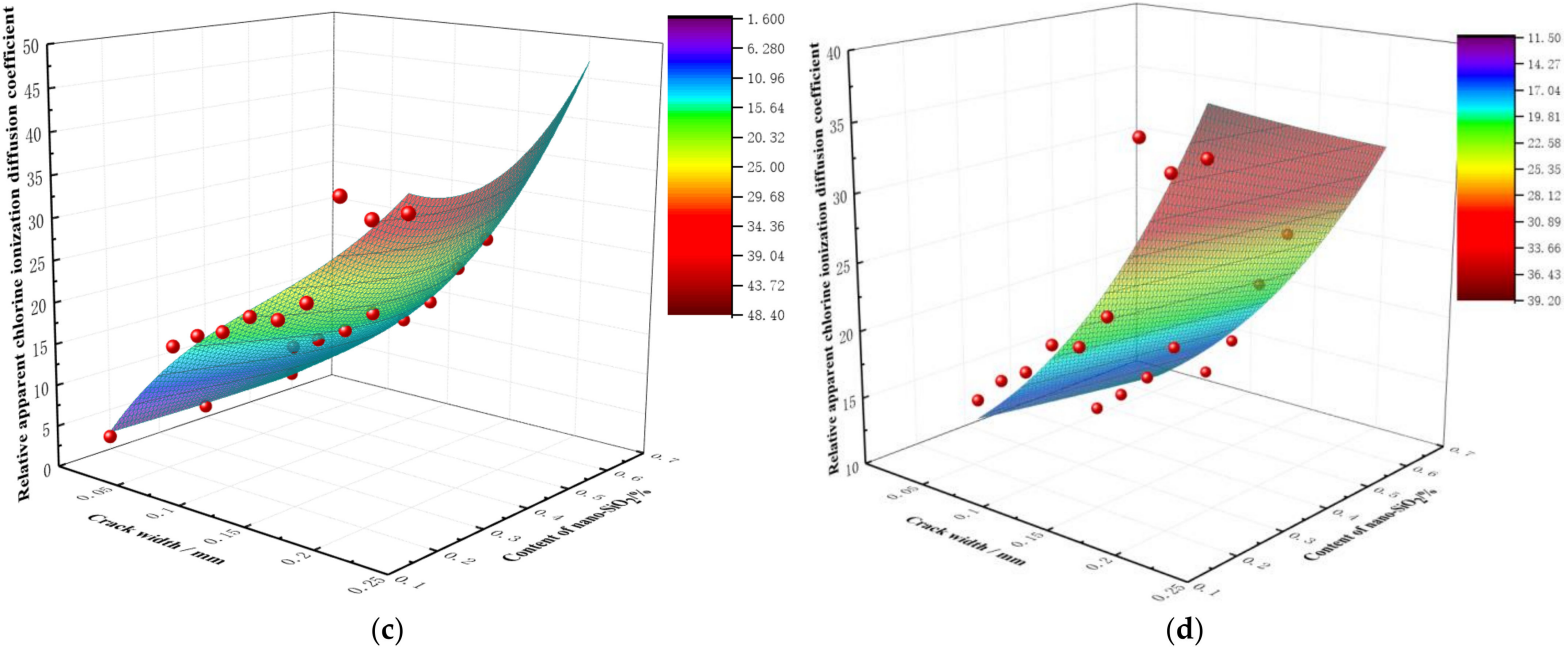
Fitting results of first power function (**a**), second power function (**b**), third power function (**c**) and exponential function (**d**).

In order to further analyze the quantitative relationship between the comprehensive effect function F(*w*) (i.e., the relative apparent chloride diffusion coefficient) and the replacement ratio of recycled aggregate (*r*), crack width (*w*) and nano-silica content (*f*), the power function and exponential function were used for a regression analysis based on the aforementioned experimental results. First, the relationship among the comprehensive effect function, the crack width and the replacement rate of recycled aggregate was fitted. Then, the relationship between the crack width and the dosage of nano-silica was analyzed by regression. The relevant results are shown in Table 6, and the partial fitting results are shown in Figure 17.

In order to further quantitatively analyze the relationship between the relative apparent chloride diffusion coefficient and the variables of crack width (*w*), replacement rate of recycled aggregate (*r*) and content of nano-silica (*f*), the Matlab software was used to carry out multiple regression analyses on the power function model. In this way, the comprehensive effect function of three factors was obtained, in which the coefficient of determination R^2^ was 0.949, indicating the adequacy of the model.

(3) $$F(w)=118.25w^{2}-0.0015r^{2}-21.85f^{2}+0.42wr+4.65wf+0.21rf+90.43w+0.12r-0.281$$

### 3.4. Sampling Results of Corroded Reinforcement

**Figure 18:**
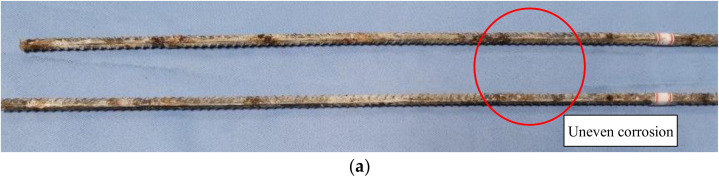

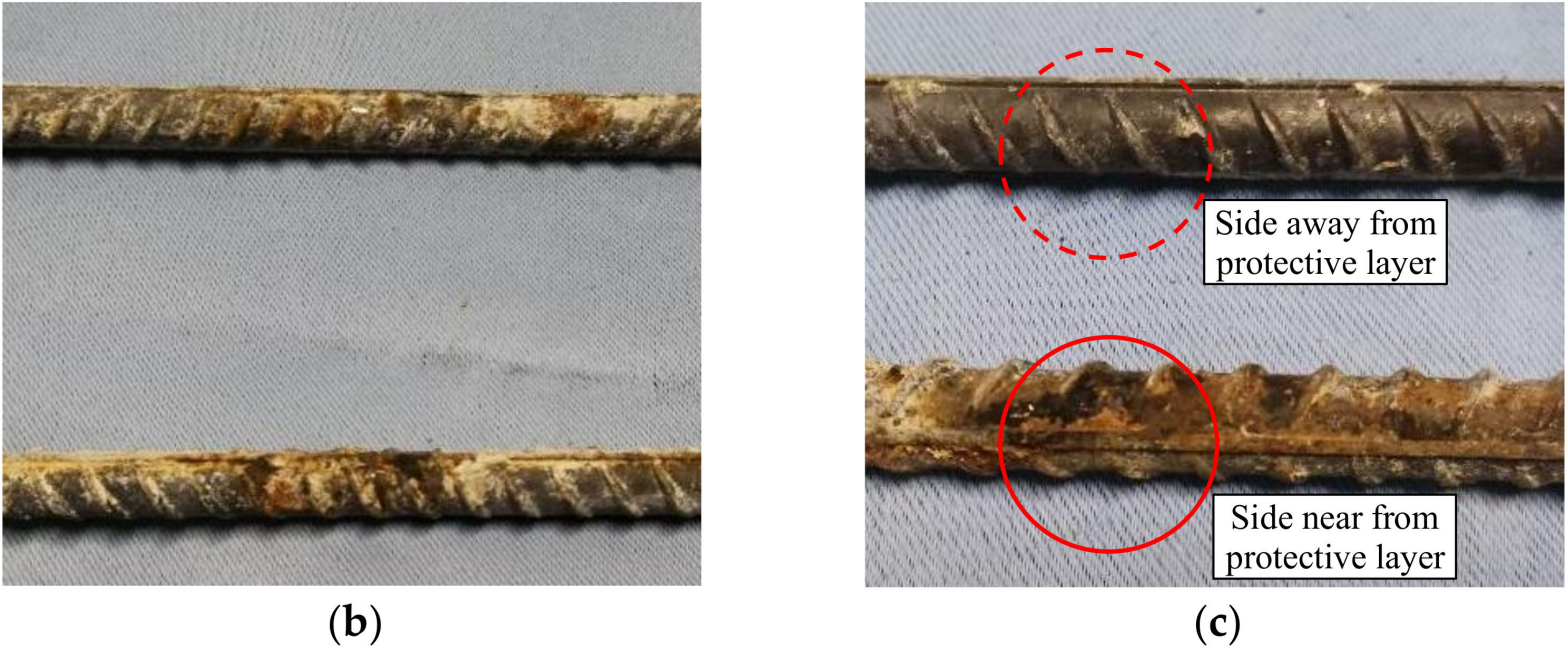
Uneven corrosion of reinforcement (**a**); Partial drawing of corroded reinforcement of No.2 beam (**b**); Contrast drawing of reinforcement corrosion near/away from protective layer (**c**).

In order to observe the corrosion of the internal reinforcements in the beam, the test beams were broken and the internal reinforcement bars were numbered. Reinforcement bar No.2 is shown in Figure 18. By observing the corrosion of these steel bars, it could be concluded that:

- In the longitudinal direction, obvious uneven corrosion occurred, as shown in Figure 18a. The main reasons for this were the influence of transverse cracks, the increased concentration of chloride ions at the cracks and the presence of water.
- The corrosion product on the side close to the concrete cover was obviously more abundant than that on the side away from the cover, indicating that uneven corrosion had occurred in the diametrical dimension of the reinforcement, as shown in Figure 18c.

## 4. Conclusions

The durability of RAC is inferior to that of natural aggregate concrete. In order to better adapt RAC to marine environments, the durability of RAC beams under a chloride ion drying-wetting cycle and continuous load was studied. On this basis, the following conclusions can be drawn:

- An effective sustained load can be provided by way of single beam self-anchoring, as used in this paper. The cracking state of a beam under external load can also be simulated. The test equipment described in this paper simplifies the resources needed for the drying-wetting cycle to a certain extent.
- In the long-term performance test, the phenomenon of self-healing was observed in the cracks. With an increase of nano-silica content, the self-healing rates of the cracks showed a tendency to first increase and then decrease. When the nano-silica content was 0.4%, the self-healing rate was optimal.
- Under the drying-wetting cycle, the variation trend of chloride ion concentration showed a wavy decline along with increasing depth; there was no obvious peak effect. The cycle ratio of drying-wetting and the effect of age on corrosion should be considered in future studies.
- When cracks appear in recycled concrete, the relative chloride ion concentration can be effectively reduced by keeping the crack width below 0.12 mm and adding about 0.2% nano-silica modified material.
- The Matlab software was used to carry out multiple regression analyses via the power function model, and to determine the functional relationships among the relative apparent chloride diffusion coefficient, the three variables of crack width (*w*), recycled aggregate replacement rate (*r*) and the content of nano-silica (*f*). For the multiple variable model, further fitting analyses with multiple basic functions are needed.

## Figures and Tables

**Table 1:** Properties of cement.

Loss on Ignition (%)	CaO (%)	SiO_2_ (%)	Al_2_O_3_ (%)	MgO (%)	Fe_2_O_3_ (%)	SO_3_ (%)	Setting Time (min)		Specific Surface Area (m^2^/kg)
							Initial	Final	
1.8	65.8	21.6	5.46	1.2	3.05	1.6	140	260	345

**Table 2:** Properties of aggregate.

Type of Aggregate	Bulk Density (kg·m^−3^)	Apparent Density (kg·m^−3^)	Water Absorption (%)
Natural aggregate	1433	2683	1.4
Recycled aggregate	1238	2562	7.2

**Table 3:** Properties of nano-silica.

Property	Value
Color	White
Purity (%)	99.8
Specific surface area (m^2^/g)	640 ± 60
Particle size (nm)	15 ± 5
pH value	6~8

**Table 4:** Mix proportion and compressive strength of recycled aggregate concrete.

No.	Consumption of Unit Volume (kg/m^3^)									Compressive Strength (MPa)
	Cement	Sand	Water	Natural Aggregate	Ordinary Recycled Aggregate	Nano Reinforced Recycled Aggregate (SiO_2_/%) *				
						0	0.2	0.4	0.6	
R0-0	361	608	195	1235	0	0	0	0	0	55.2
R25-0	361	608	195	923.2	308.8	0	0	0	0	54.9
R50-0	361	608	195	617.5	0	617.5	0	0	0	56.1
R50-2	361	608	195	617.5	0	0	617.5	0	0	56.6
R50-4	361	608	195	617.5	0	0	0	617.5	0	59.8
R50-6	361	608	195	617.5	0	0	0	0	617.5	58.2

* The content of nano-silica accounts for the mass percentage of the gelling material.

**Table 5:** Statistics of self-anchored cracks in the test beam (unit: mm).

No.	Control Width	Actual Maximum Width	Actual Minimum Width	Average Width	Minimum Spacing	Maximum Spacing	Average Spacing	Total Crack Number
R0-0-0.2 *	0.2	0.158	0.055	0.093	32	122	81.8	8
R25-0-0.2	0.2	0.181	0.036	0.097	50	138	90.0	8
R50-0-0.2	0.2	0.137	0.075	0.099	49	118	78.9	8
R50-2-0.1	0.1	0.095	0.055	0.091	79	123	100.7	7
R50-2-0.2	0.2	0.181	0.075	0.130	52	133	92.3	8
R50-2-0.3	0.3	0.227	0.075	0.159	67	103	75.6	9
R50-4-0.2	0.2	0.158	0.055	0.092	49	118	74.8	9
R50-6-0.2	0.2	0.158	0.095	0.121	65	98	82.9	8

* R0-0-0.2 indicates that the replacement rate of recycled aggregate is 0%, the content of nano-silica is 0, and the control crack width is 0.2 mm.

**Table 6:** Parameters of each fitting model.

Independent Variable	Type of Function	Highest Power	Function Model	*R* ^2^
*w*, *r*	Power function	First power	F(w)=120.5w+0.07r−1.25	0.956
		Second power	$F(w)=176.1w^{2}-0.0019r^{2}+0.38wr+81.6w+0.13r-0.141$	0.966
		Third power	$\begin{array}{ll} F(w) & =5211.4w^{3}+0.3r^{3}-13.8w^{2}r-0.01wr^{2}\\ & -720.9w^{2}-19.9r^{2}+3.4wr+93.6w+333.2r+0.94 \end{array}$	0.976
*w*, *f*	Power function	First power	F(w)=146.4w−9.8f+5.6	0.941
		Second power	$F(w)=8.1w^{2}+57.6f^{2}-18.1wf+135.3w-52.2f+12.1$	0.937
		Third power	$\begin{array}{ll} F(w) & =2344.6w^{3}+4.9f^{3}+1827.4w^{2}f+546.9wf^{2}\\ & -1253.1w^{2}-2.4f^{2}-743.3wf+351.6w-1.3f+2.3 \end{array}$	0.930
	Exponential function *	-	$F(w)=9.7e^{6.1w-0.4f}$	0.919

* The exponential function was not suitable for the fitting between crack width and replacement rate; the point where the crack width was 0 had to be discarded when fitting the exponential function.
